# Hiatus Hernia Repair with Bilateral Oesophageal Fixation

**DOI:** 10.1155/2015/693138

**Published:** 2015-04-30

**Authors:** Rajith Mendis, Caran Cheung, David Martin

**Affiliations:** ^1^Westmead Hospital, Sydney, NSW 2145, Australia; ^2^University of Sydney, Sydney, NSW 2006, Australia; ^3^Department of Upper GI Surgery, Concord Hospital, Sydney, NSW 2139, Australia; ^4^Department of Upper GI Surgery, Royal Prince Alfred Hospital, Sydney, NSW 2050, Australia; ^5^Department of Upper GI Surgery, Strathfield Private Hospital, Sydney, NSW 2135, Australia

## Abstract

*Background*. Despite advances in surgical repair of hiatus hernias, there remains a high radiological recurrence rate. We performed a novel technique incorporating bilateral oesophageal fixation and evaluated outcomes, principally symptom improvement and hernia recurrence. *Methods*. A retrospective study was performed on a prospective database of patients undergoing hiatus hernia repair with bilateral oesophageal fixation. Retrospective and prospective quality of life (QOL), PPI usage, and patient satisfaction data were obtained. Hernia recurrence was assessed by either barium swallow or gastroscopy. *Results*. 87 patients were identified in the database with a minimum of 3 months followup. There were significant improvements in QOL scores including GERD HRQL (29.13 to 4.38, *P* < 0.01), Visick (3 to 1), and RSI (17.45 to 5, *P* < 0.01). PPI usage decreased from a median of daily to none, and there was high patient satisfaction (94%). 57 patients were assessed for recurrence with either gastroscopy or barium swallow, and one patient had evidence of recurrence on barium swallow at 45 months postoperatively. There was an 8% complication rate and no mortality or oesophageal perforation. *Conclusions*. This study demonstrates that our technique is both safe and effective in symptom control, and our recurrence investigations demonstrate at least short term durability.

## 1. Introduction

Achieving durable hernia repair and symptom outcomes whilst minimising untoward sequelae are key challenges in hiatus hernia surgery. The technique for repair of the hiatus hernia has evolved significantly from the original approach described in 1919 by Soresi detailing reduction of the hernia with closure of the crus [1]. Current methods favour complete mobilisation with division of adhesions, fundoplication (often around an oesophageal bougie), and crural closure [2]. There remain, however, many variations on this technique and there is no one standardised method which has been proven to be superior.

One of the major issues related to hiatus hernia repair is hernia recurrence, with rates of recurrence varying from 4 to 42% at intermediate followup [3–5]. This rate may deteriorate in the longer term with a more recent study reporting a high radiological recurrence rate of 66% in 35 patients undergoing barium studies at a median followup of 99 months [6], suggesting concerns about the longevity of current repairs. However, despite this higher rate, quality of life (QOL) assessments were not significantly affected and only one patient required reoperation.

Techniques to decrease recurrence and maximise outcomes have been investigated in the literature and include the use of meshes and gastropexy and the degree and type of fundoplication.

Use of nonabsorbable mesh has been associated with improved rates of recurrence [7] but can be associated with local mesh related complications including erosion, hiatal stenosis, and fibrosis [8]. Biologic meshes have been associated with decreased rates of complications with respect to nonabsorbable meshes [9] and short term efficacy [10, 11] but long term studies are awaited.

Partial and full (360°) fundoplication have been shown in meta-analyses to have similar control of reflux symptoms, with similar satisfaction and reoperative rates. The partial fundoplication group generally has less dysphagia and bloating but a slightly higher attrition rate for reflux with longer followup [12]. One meta-analysis has shown a higher reoperation rate in the total fundoplication group, predominately due to dysphagia [13].

Gastropexy may have some role in reducing postoperative recurrence [14], but bilateral oesophageal fixation as performed in our series has not previously been in any of the randomised control trials evaluating fundoplication techniques.

We performed a novel technique involving bilateral oesophageal fixation to the oesophageal crura coupled with a fixed anterior fundoplication in an effort to reduce the risk of recurrence. Our study evaluates the outcomes using this technique, principally symptom improvement and hernia recurrence.

## 2. Materials and Methods

### 2.1. Technique

Our technique involves laparoscopic optical entry, with standard circumferential mobilisation of the phrenooesophageal ligament, followed by dissection and reduction of the peritoneal sac from the mediastinum. The oesophagus is then mobilised high into the mediastinum and the crura is closed with deep interrupted anterior and posterior sutures to maintain the oesophagus in the mid-crura, using a nonabsorbable braided suture (0 Ethibond, Ethicon). A 56 French bougie is used to calibrate the closure.

**Figure 1:**
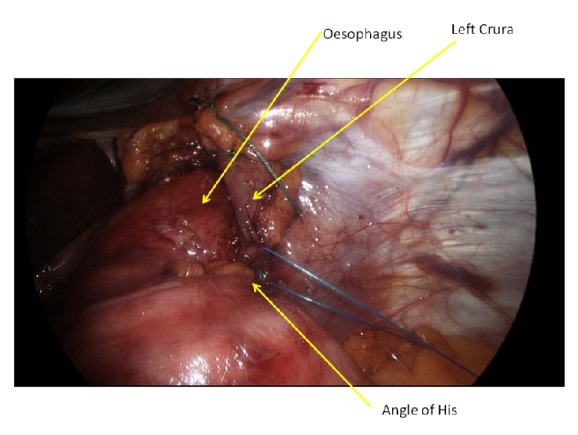
Left sided fixation suture with oesophagus and angle of His anchored to left crura.

**Figure 2:**
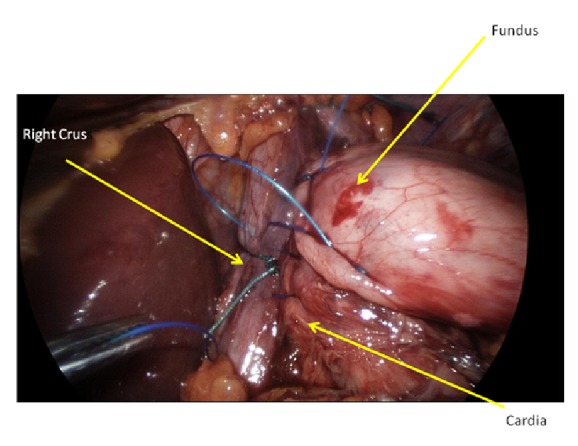
Right sided fixation suture involving running bites along entire length of wrap.

Left sided fixation of the oesophagus to the crura is then performed, centred at the 3 o'clock position with a figure of 8 suture, with the same suture used to then incorporate the “angle of His” prior to tying (Figure 1). The anterior wrap with right sided oesophageal fixation is then performed with a running suture encompassing the fundus, right oesophagus, and crura, from the 11 o'clock position anteriorly to the 7 o'clock position posteriorly over 3-4 bites (Figure 2). The anterior wrap is then further sutured to the hiatus apex with a figure of 8 suture, including the anterior oesophagus (12 o'clock position) only if the anterior vagus nerve can be identified and excluded from the suture. A nonabsorbable nonbraided monofilament suture (Novafil, Covidien) is used for all fixation sutures.

Absorbable biological mesh (Surgisis, Cook Medical) is infrequently used on the posterior crus and only if there is marked tension at closure.

This technique has evolved from the phrenooesophageal ligament repair (described by Nathanson) though instead of repairing the damaged structure, we decided to directly fix the two organs being held by it.

By performing an anterior wrap, we were able to fix the fundus to the oesophagus along almost the entire length of the wrap, thereby theoretically decreasing the risk of migration of the wrap into the chest. This form of fixation, essentially across the anterior 180 degrees of the hiatus, involving oesophagus, fundus, and crura, has not been explicitly described previously. Certainly, the incorporation of the left sided fixation of oesophagus and angle of His to the left crura does not seem to be a feature of anterior wraps used in the randomised controlled trials.

All operations were performed under the auspices of the senior author (David Martin) with the senior author scrubbed and either performing the surgery or assisting senior surgical Upper GI fellows.

### 2.2. Study Design

A retrospective study was performed on a prospectively collected electronic database of patients who had hiatus hernia repair with the abovementioned oesophageal fixation technique, performed by the same surgeon. All patients underwent routine gastroscopy and manometry. 24 hour pH studies were usually performed, if tolerated and nuclear medicine isotope scans were also often performed. The 24-hour pH studies were considered positive if the patients had a pH < 4 for greater than 5% of the time. Many patients also had fluoroscopic swallow studies. Some patients with large symptomatic hiatus hernias did not undergo assessment with nuclear medicine isotope scans or 24-hour pH monitoring.

Retrospective and prospective QOL, Proton Pump Inhibitor (PPI) usage, and patient satisfaction (3-point scale) data were obtained with phone interviews. QOL was assessed using the validated gastrooesophageal reflux disease health related quality of life (GERD HRQL, 10-question, 5-point scale) [15], Visick score (4-point symptom scale) and the Reflux Symptom Index (RSI, 9-question, 5-point scale assessing laryngopharyngeal reflux) [16]. PPI usage was assessed according to frequency.

All patients were contacted postoperatively for preoperative and postoperative data. Some patients had prospectively completed the preoperative assessment.

Hernia recurrence was assessed by either barium swallow or gastroscopy. As part of routine postoperative followup most patients underwent a 6-month gastroscopy. All patients, not having undergone a postoperative gastroscopy within 5 months of their phone interview were also invited to have a barium swallow exam assessing for hiatus hernia recurrence using an established protocol encompassing views of the lower oesophagus, supine fundus, right lateral decubitus fundus, and erect lateral. The reports of any recent gastroscopy were also reviewed for any sign of recurrence. Recurrence was assessed at 6 months or greater postoperatively.

### 2.3. Statistical Analysis

Data was analysed using SPSS for statistical analysis, with the Wilcoxon Signed-Ranks test used to compare non-normally distributed results, confirmed with the Shapiro-Wilk test.

### 2.4. Ethical Statement

The project and data collection was approved by Sydney Local Health District Human Research Ethics Committee covering the associated hospitals and as accredited by the NSW Ministry of Health (File Ref: LNR/13/CRGH/194).

## 3. Results

93 consecutive patients underwent laparoscopic hiatus hernia repair between 2008 and 2012 of which 87 had a minimum of 3 months followup. There were 36 (39%) male and 57 (61%) female patients, with a mean age of 61 (range 24–89). 7 were with recurrent hiatus hernias following previous fundoplication. 46 patients had preoperative 24-hour pH studies and 50 patients had preoperative isotope studies, 31 patients having both. There were no conversions to open surgery, no oesophageal perforations, and no mortality. Absorbable mesh (Surgisis, Cook Medical) was used in 10 patients (11%). There were 6 complications (8%): 1 small volume bile leak in drain following liver laceration from a liver retractor which settled with conservative management, 2 patients with postoperative chest pain, 1 patient with a food bolus obstruction on day 2 postoperatively after inadvertently being started on a full diet, and 2 patients with diarrhoea immediately postoperatively.

**Figure 3:**
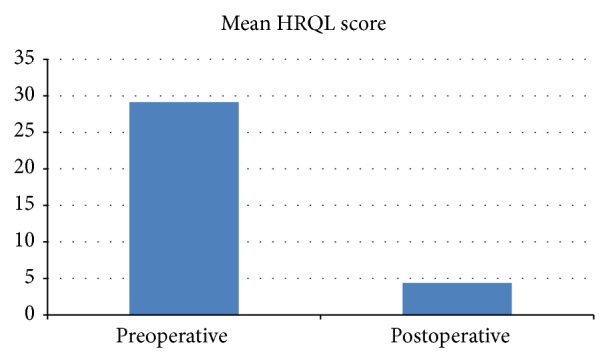
Comparison of preoperative and postoperative gastrooesophageal reflux QOL scores.

**Figure 4:**
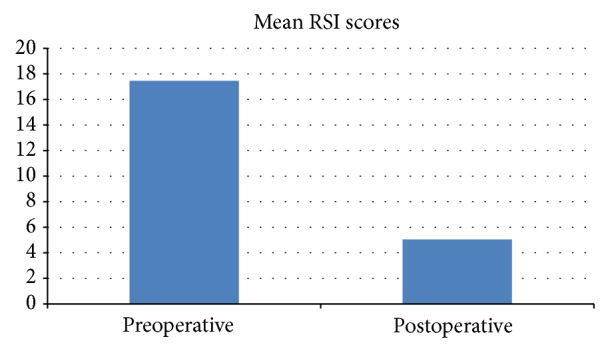
Comparison of preoperative and postoperative laryngopharyngeal reflux QOL scores.

Quality of life (QOL) data was obtained from 56 patients, with 36 (66%) of the preoperative assessments being completed retrospectively. The postoperative QOL data was obtained at a mean of 24 months postoperatively (range 3–48 months). Mean HRQL scores improved from 29.13/50 preoperatively to 4.38/50 postoperatively (*P* < 0.001) (Figure 3). RSI scores improved from a mean of 17.45/45 preoperatively to 5.04 postoperatively (*P* < 0.001) (Figure 4) and the number of RSI scores above 13 (considered positive for LPR) decreased from 32 preoperatively to 9 postoperatively.

There was also a significant improvement in Visick scores from a median of 3 preoperatively to 1 postoperatively (*P* < 0.001).

PPI usage decreased from a median of daily to none postoperatively (*P* < 0.001), however 15 patients (27.3%) were still using a PPI daily postoperatively (Table 1).

Satisfaction scores were obtained from 53 patients with 50 (94%) being satisfied, 1 (2%) neutral, and 2 (4%) dissatisfied.

**Figure 5:**
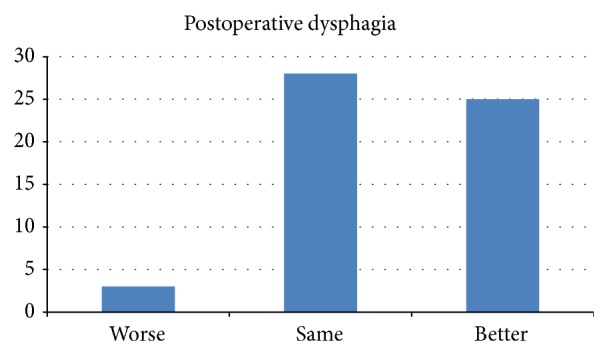
Postoperative dysphagia.

Dysphagia scores were analysed pre- and postoperatively and only 3 patients (5%) had increases in dysphagia scores postoperatively. Two patients increased from 0 to 1/5, pre- and postoperatively, and the other from 0 to 3/5 in severity. 28 patients had no difference in dysphagia and 25 patients experienced improvement in dysphagia (Figure 5).

57 patients had either follow-up gastroscopy or fluoroscopy for investigation of recurrence at least 6 months postoperatively, with a mean followup of 17.8 months (range 6–49 months). Gastroscopy was the most recent investigation in 33 patients (58%) and barium swallow study in 24 (42%). Of these patients, 1 patient (1.8%) had a recurrence on a followup barium swallow study performed at 45 months postoperatively. This patient had undergone repair of a massive hernia containing 100% of the stomach and had some recurrent reflux symptoms which were controlled with a regular PPI. When looking at patients with more than 18 months followup, there were 21 patients, with a mean followup of 33.2 months, with the single recurrence (5%).

## 4. Discussion

Laparoscopic hiatus hernia repair has been proven to be safe and effective, but the recurrence rate remains not insignificant. The method of bilateral oesophageal fixation utilised with our repair has been performed in attempt to prevent recurrence, specifically from wrap migration and telescoping phenomenon. The incorporation of the angle of His into this fixation was hoped to add further effect to the anterior wrap, plus gastric fixation, thereby hopefully decreasing the attrition of reflux improvement seen in some partial fundoplication studies whilst attempting to maintain the benefits of decreased side effects of dysphagia and bloating seen with the 360-degree wrap. In our series, absorbable mesh was used in 11% of repairs, and only when there was marked tension. We used absorbable mesh to reduce the risk of complications associated with nonabsorbable mesh. The mesh used at the time of the study was an accepted standard though we have since changed to more robust Biomesh. We have analysed data from our database of patients undergoing this novel technique to assess safety, efficacy, and durability.

With 87 patients included in the study and no mortality or oesophageal perforations, it appears that this technique is safe, though we would advise close adherence to correct techniques as described in the following, particularly in suturing the oesophagus and wrap to avoid enteric injury. The complication rate of 8% is comparable to other studies [6, 7].

*Surgical Techniques to Avoid Enteric Tears with Oesophageal Fixation Sutures*

1. No tension;
2. monofilament suture;
3. single smooth passage of suture through oesophagus with each bite;
4. minimal traction on the oesophagus by the assistant once fixation is underway.

Initial quality of life data demonstrates that this hernia repair provides very good symptom improvement for both classical heartburn and laryngopharyngeal symptoms, with minimal dysphagia, and resultant high levels of patient satisfaction.

Despite the QOL score improvement, however, a moderate percentage of patients (27.3%) were still using a PPI postoperatively and this remains an area of concern. This is not uncommon, though, with long term studies showing continued use of PPIs in up to 62% of patients having antireflux operations [17]. This may be attributed to previous positive experiences with a PPI preoperatively, resulting in a predilection towards restarting PPI therapy following onset of mild or atypical symptoms. The rate of true reflux as measured by pH monitoring in patients requiring postoperative PPIs has been reported as 26% [18]. The high number of negative studies highlights the need for objective followup and investigation prior to considering any revisional surgery in these patients.

Our study identified one recurrence although the patient's symptoms were controlled with medical therapy. Recurrence was assessed with either a barium swallow study or a gastroscopy. The barium swallow study utilised a defined protocol to maintain the reliability of the study, although a potential for error exists as the films were reviewed and reported by a variety of radiologists. However these radiologists were not aware of the novel technique utlitised in the repair of the hiatus hernia. A potential bias exists in the patients assessed for recurrence with a gastroscopy as most were conducted by the surgical teams at the Upper GI departments at the participating campuses of the senior author and primary surgeon.

## 5. Conclusion

At intermediate followup, with only one hiatus hernia recurrence in our study population at 45 months, there is potential that this technique may provide improved durability of the hernia repair. With further followup of this growing cohort it will be interesting to further investigate the mode of recurrence and the role of wrap migration or telescoping phenomenon, which we have attempted to prevent by both the oesophageal and anterior wrap fixation.

We believe this technique of hiatus hernia repair offers safe and potentially durable outcomes, with a low likelihood of untoward side effects, and high patient satisfaction.

## Figures and Tables

**Table 1:** Comparison of preoperative and postoperative PPI usage.

	Preoperative	Postoperative
None	8	33
Less than 1/week	1	6
1–3x/week	1	1
4–6x/week	1	0
Daily	44	15
